# The effects of cervical cerclage on the vaginal microbiota and its metabolites in pregnant women

**DOI:** 10.3389/fcimb.2025.1577947

**Published:** 2025-06-16

**Authors:** Jun Zhang, Lihua Wang, Mengjun Zhang, Zhimin Xu, Yijing Zheng, Bingqing Lv, Mian Pan

**Affiliations:** Department of Obstetrics & Gynecology, Fujian Maternity and Child Health Hospital, College of Clinical Medicine for Obstetrics & Gynecology and Pediatrics, Fujian Medical University, Fuzhou, Fujian, China

**Keywords:** cervical cerclage, pregnant women, clinical characteristics, vaginal microbiota, vaginal metabolites

## Abstract

Cervical cerclage is widely used to reduce the risk of preterm birth in pregnant women. The effects of cervical cerclage on the vaginal microbiota and its metabolites are not fully clear. The purpose of this study was to explore the influence of cervical cerclage on the vaginal microbiota and its metabolites. Our results showed that the clinical characteristics [white blood cell (WBC), neutrophil, lymphocyte, monocytes, platelet, NLR, PLR, SII, SIRI, and C-reactive protein (CRP)] and α-diversity (Observed, Shannon, Chao1, and Simpson indexes) of the vaginal microbiota were not altered during pregnancy after cervical cerclage. 16S rRNA gene sequencing found that the relative abundance of *Muribaculaceae* and *Blautia* was significantly increased in the post-cerclage group compared with the pre-cerclage group, but the relative abundance of *Sneathia* was significantly reduced. In addition, the volcano plot revealed that a total of 19 metabolites [including alpha-hydroxyalprazolam, LPE (18:1(9Z)/0:0), PS(16:0/15:0), *N*-acetylhistamine, carnitine, pseudouridine, and allopregnanolone] were significantly changed during pregnancy after cervical cerclage. Pathway analysis based on the Kyoto Encyclopedia of Genes and Genomes (KEGG) database showed that the changes in the vaginal microbiota and its metabolites mainly involved purine metabolism and amino acid metabolism. The alteration in the vaginal microbiota and its metabolites induced by cervical cerclage is associated with the therapeutic efficacy of cervical cerclage. Further studies are needed to explore how the vaginal microbiota affects the outcomes of pregnancy.

## 1 Introduction

Preterm birth is described as birth prior to 37 weeks of gestation (or less than 259 days from the first day of a woman’s last menstrual period), including spontaneous and iatrogenic preterm births (Wang et al., 2024). Preterm birth is a major factor in infant morbidity and mortality worldwide, and is strongly related to long-term adverse outcomes in children (Liao et al., 2023). The high incidence of preterm birth not only increases the annual societal economic burden but also seriously influences family happiness and social harmony. According to the Global Disease Burden Study, more than 15 million babies are born premature every year, and the prevalence of preterm birth is continuously increasing (Elkhouli et al., 2024). Among these, approximately 45% of premature infants are diagnosed with spontaneous preterm labor with intact membranes, and approximately 30% of premature infants worldwide are diagnosed with spontaneous preterm labor with ruptured membranes (Beernink et al., 2023). According to a previous report, the occurrence of preterm birth is associated with a short cervix, region, extremes of maternal age (<25 years and >35 years) and body mass index (BMI < 18 and BMI > 28), low socioeconomic status, smoking, and genetic polymorphisms (Wei et al., 2024). For example, it is reported that the rates of preterm birth accounted for more than 80% of global cases in low-income and middle-income countries, such as Southern Asia and Sub-Saharan Africa (Ohuma et al., 2023). At present, cervical cerclage is widely used as a surgical intervention for incompetent cervix that saves premature fetuses and reduces the risk of adverse pregnancy outcomes (Seyama et al., 2022). Despite the huge advancement in cervical cerclage, there was an obvious difference in the therapeutic efficacy of cervical cerclage among different individuals. However, there has been no systematic study that explored the association between cervical cerclage and therapeutic efficacy.

As is well known, the vagina is home to a rich and complex community of microbes, including bacteria, fungi, viruses, and archaea. *Lactobacillus* is the most abundant microorganism in the vagina of healthy individuals, such as *L. crispatus*, *L. gasseri*, *L. iners*, or *L. jensenii (* Hong et al., 2021 *)*. According to the high relative abundance of specific *Lactobacillus* species, the vaginal microbiota is classified into five community state types (CSTs), namely, CST I, CST II, CST III, CST IV, and CST V (Chen et al., 2024). Regardless of the CST, *Lactobacillus* species constitute a dominant and essential component of the vaginal microbiota. These *Lactobacillus* species are beneficial for maintaining the acidity of the vagina by producing some organic acids (including short-chain fatty acids and lactic acid), which prevent the growth and reproduction of harmful bacteria. It is reported that vaginal microbial communities regulate homeostasis and have a significant influence on reproductive health. For example, there was more stability throughout the entire pregnancy in pregnant women with a higher abundance of vaginal *Lactobacillus* spp., which is associated with positive pregnancy outcomes (Schuster et al., 2024). In contrast, the dysbiosis of the vaginal microbiota mainly manifested as a reduction of *Lactobacillus* species abundance and an increase in diversity of other bacteria, such as *Gardnerella*, *Prevotella cluster* 2, *Lachnospiraceae*, *Sneathia amnii*, and *Saccharibacteria (* Peelen et al., 2019). A previous report suggested that the vaginal microbiota of pregnant women who ultimately had spontaneous preterm birth were dominated by *L. iners* and the subspecies clade of *Gardnerella vaginalis (* Fettweis et al., 2019). Therefore, vaginal microbial community balance is an essential factor that reduces the risk of preterm birth. However, whether cervical cerclage reduces the risk of premature birth by altering the composition of the vaginal microbiota needs to be explored.

The aim of this study was to explore whether the dysbiosis in the vaginal microbial community is associated with the failure of cervical cerclage, thus making preventative treatment accessible to all.

## 2 Methods and materials

### 2.1 Participants

**Figure 1:**
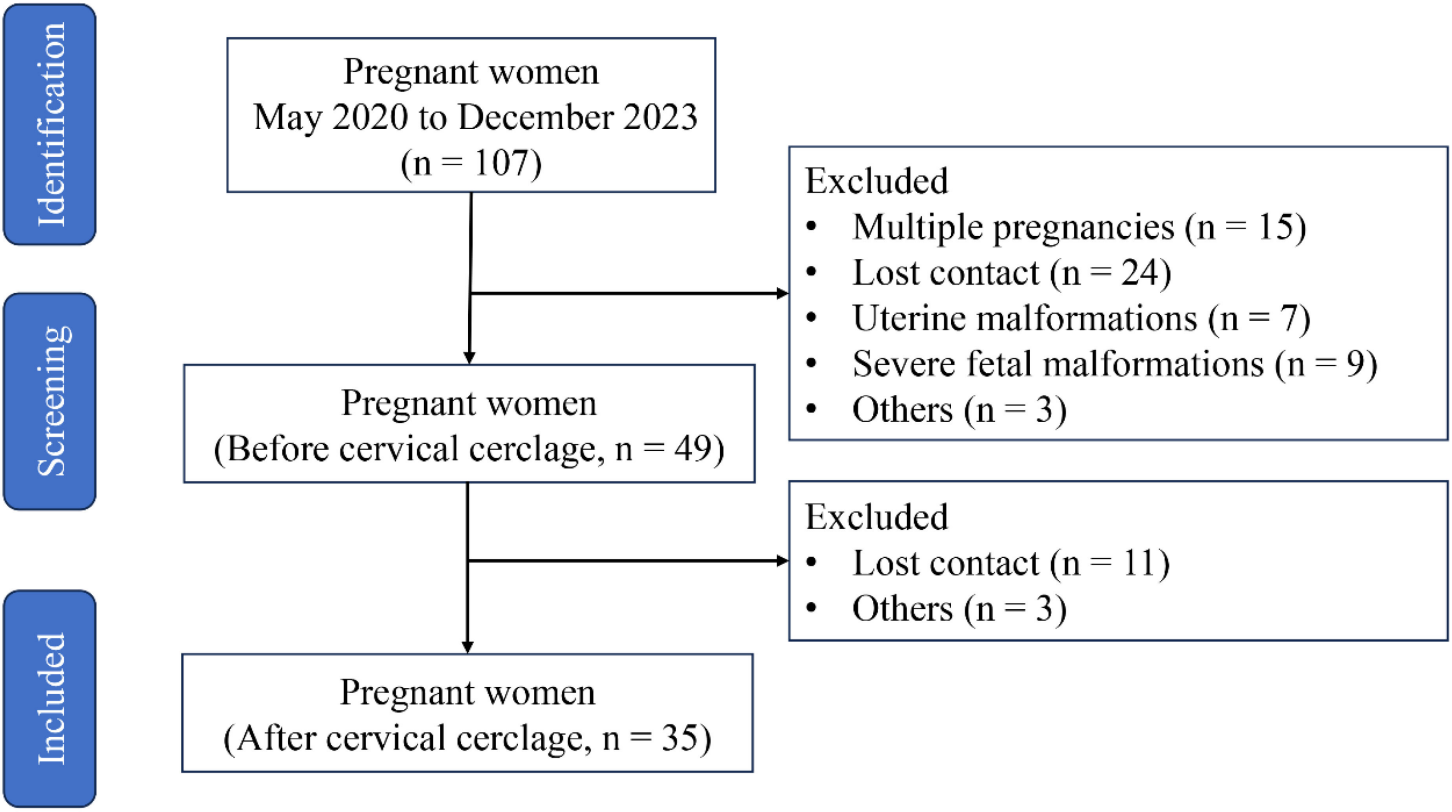
Flowchart of the study population.

This prospective observational study was carried out at the Fujian Maternity and Child Health Hospital (no. 2020-1-024), and clinical data and neonatal outcomes from patients with cervical insufficiency who underwent cervical cerclage between 1 May 2020 and 30 September 2023 were collected. The recruitment criteria are as follows: (1) age from 21 to 35 years; (2) BMI from 18 to 28; (3) gestational age from 18 to 24 weeks; (4) singleton pregnancy; (5) cervical dilation of ≥1 cm; (6) complete membrane; and (7) no vaginal bleeding. Patients with the following conditions have been excluded: (1) labor onset; (2) membrane injury; (3) clinical chorioamnionitis, manifested as a temperature of more than 38.0°C, heart rate of more than 100 beats/min, fetal heart rate of more than 160 beats/min, uterine tenderness, malodorous vaginal discharge, and abnormal peripheral blood leukocyte count (WBC count of more than 15 × 10^9^/L or left shift in neutrophils); (4) placental abruption; (5) fetal congenital anomalies or maternal medical or surgical complications requiring termination of pregnancy; and (6) twin or multiple pregnancies. In the end, 49 patients met the recruitment requirements, but only 35 patients had complete medical record data after cervical cerclage for 6 weeks (**Figure 1**).

### 2.2 Cervical cerclage procedure

The cervical cerclage procedure was performed using the McDonald technique with a braided Mersilene 5-mm tape. All patients were in the Trendelenburg position under spinal anesthesia to minimize pressure on the fetal membranes. The membranes were gently retracted into the internal os using a sponge stick or Foley balloon after the cervical lips were delicately grasped with Allis forceps. Sutures were then placed near the vaginal–cervical junction. Once the suture effectively closed the endocervical canal, alternating knots were meticulously tied to ensure secure closure and prevent slippage.

### 2.3 Sample collection

Before cervical cerclage, the blood and cervicovaginal lavage samples were collected. In the 6th week after cervical cerclage, the blood and cervicovaginal lavage samples were re-collected. Among them, cervicovaginal lavage samples were collected according to our previous study (Li et al., 2023). WBCs, neutrophils, lymphocytes, monocytes, platelets, and CRP in the blood were detected using a Beckman Coulter AU5822 analyzer. The cervicovaginal lavage samples were stored at −80°C until used.

### 2.4 16S rRNA gene sequencing

The sequencing analysis of the vaginal microbiota was implemented by the MiSeq platform based on the methodology outlined in a previous report with some modifications. In brief, total bacterial DNA from the CVF sample was extracted using a commercially available total DNA extraction kit (MoBio, Carlsbad, CA, USA), and then the V3–V4 regions of bacterial 16S rRNA genes were amplified by broad-range bacterial primers, namely, 338F primers (5′-CCTAYGGGRBGCASCAG-3′) and 806R primers (5′-GGACTACHVGGGTWTCTAAT-3′). These products were purified using 2.0% agarose gel electrophoresis; target fragment was collected and recovered by the Agencourt AMPure XP Kit (Hangzhou, China). The content of each sample was detected by a Nanodrop 2000 spectrophotometer (Thermo Fisher Scientific, CA, USA). Sequencing libraries consisted of equal concentrations of each sample, and their quality was evaluated on the Qubit@ 2.0 Fluorometer (Thermo Fisher Scientific, CA, USA) and then was implemented on the Illumina Miseq platform (San Diego, CA, USA) at Shanghai Biotree Biotech. Co., Ltd.

The raw data were filtered, denoised, and merged; chimera was removed using Microbial Ecology software (v 2.0); and the high-quality sequences were collected and then grouped into operational taxonomic units (OTUs) with similarities of more than 97%. The taxonomy annotation process was carried out on the OTU sequences using the Mothur approach and the SSU rRNA database of SILVA138.1. α-diversity and β-diversity of the vaginal microbiota were analyzed by X shell (v 7.0). The overall differences in the vaginal microbiota were assessed based on the principal coordinates analysis (PCoA) by R software (v 4.4.2), and the key microbial phylotypes were screened using the Microbial Ecology software.

### 2.5 Metabolomics analysis

The vagina was detected by a single-use sterile endoscope, and the vaginal wall was gently rotated by a cotton swab. The sampling loop was taken out from the sheath after reaching the uterus, and rotated 15 times to collect the endometrial sample, which is beneficial for eliminating cross-contamination between the intrauterine and vaginal samples. Metabolomic profiles of cervicovaginal lavage samples were collected using a UPLC-Q-TOF/MS equipped with Thermo Q Exactive mass spectroscopy. The samples were separated with an ACQUITY UPLC^®^ HSS T3 (150×2.1 mm, 1.8 μm, Waters) column maintained at 40°C. Mass spectrometry (MS) was carried out by a Xevo G2-XS QTOF mass spectrometer (ACQUITY, WATERS, USA) in both ESI+ and ESI− modes. Raw data were converted into mzXML format using the Proteowizard software (v3.0.8789) and carried out in the XCMS program. The normalized data were subjected to multivariate statistical analysis in MetaboAnalyst 6.0, including principal component analysis (PCA), partial least squares discriminant analysis (PLS-DA), and orthogonal projections to latent structures discriminant analysis (OPLS-DA). The differential metabolites were screened based on prediction (VIP) > 1, fold change >2 or < 0.5, and *p* < 0.05. Meanwhile, the differential metabolites related to metabolic pathways were analyzed and visualized based on the Kyoto Encyclopedia of Genes and Genomes (KEGG) database.

### 2.6 Statistical analysis

All data were presented as mean ± SD using the GraphPad Prism 7.0 software (California, USA), and ^*^ *p* < 0.05 or ^**^ *p* < 0.01 suggests statistical significance compared to the pre-cerclage group.

## 3 Results

### 3.1 Effects of cervical cerclage on the clinical characteristics of patients

**Figure 2:**
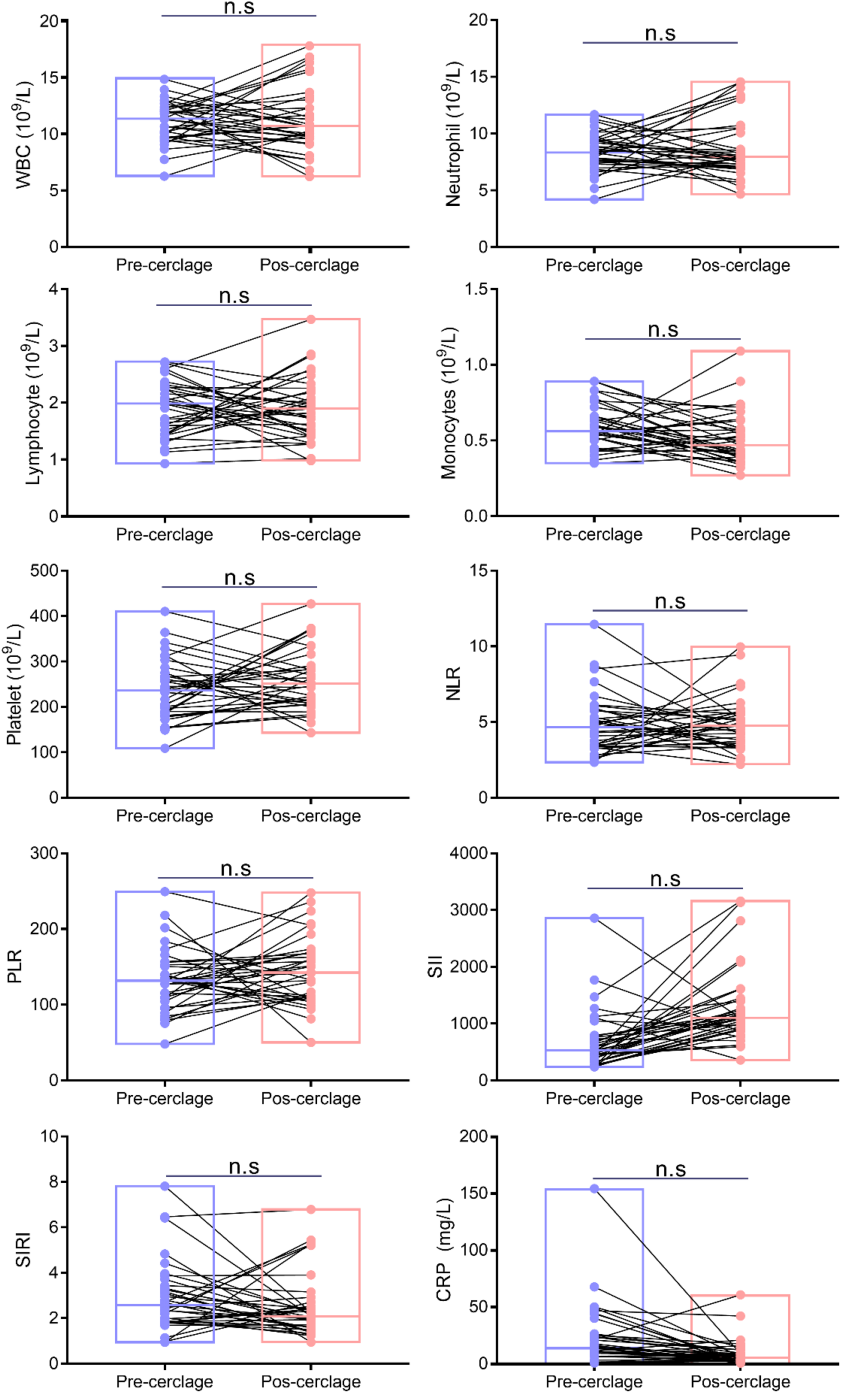
Comparisons of maternal serum inflammatory biomarkers. ns, not significant.

There was no significant difference in WBC (from 11.05 ± 1.81 to 11.31 ± 2.94 10^9^/L), neutrophils (from 8.40 ± 1.69 to 8.77 ± 2.67 10^9^/L), lymphocytes (from 1.88 ± 0.49 to 1.90 ± 0.55 10^9^/L), monocytes (from 0.60 ± 0.14 to 0.51 ± 0.17 10^9^/L), platelets (from 237.09 ± 67.03 to 259.31 ± 67.96 10^9^/L), neutrophil-to-lymphocyte ratio (NLR) (from 4.85 ± 1.99 to 4.88 ± 1.73), platelet-to-lymphocyte ratio (PLR) (from 131.62 ± 41.44 to 144.54 ± 44.93), systemic immune inflammation index (SII) (from 689.23 ± 525.32 to 1,285.20 ± 656.16), systemic inflammation response index (SIRI) (from 2.94 ± 1.54 to 2.54 ± 1.41), and CRP (from 23.45 ± 27.22 to 9.03 ± 11.89 mg/L) between before and after cervical cerclage (*p* > 0.05), indicating that cervical cerclage did not affect the clinical characteristics of patients (**Figure 2**).

### 3.2 Effects of cervical cerclage on the vaginal microbial community of patients

**Figure 3:**
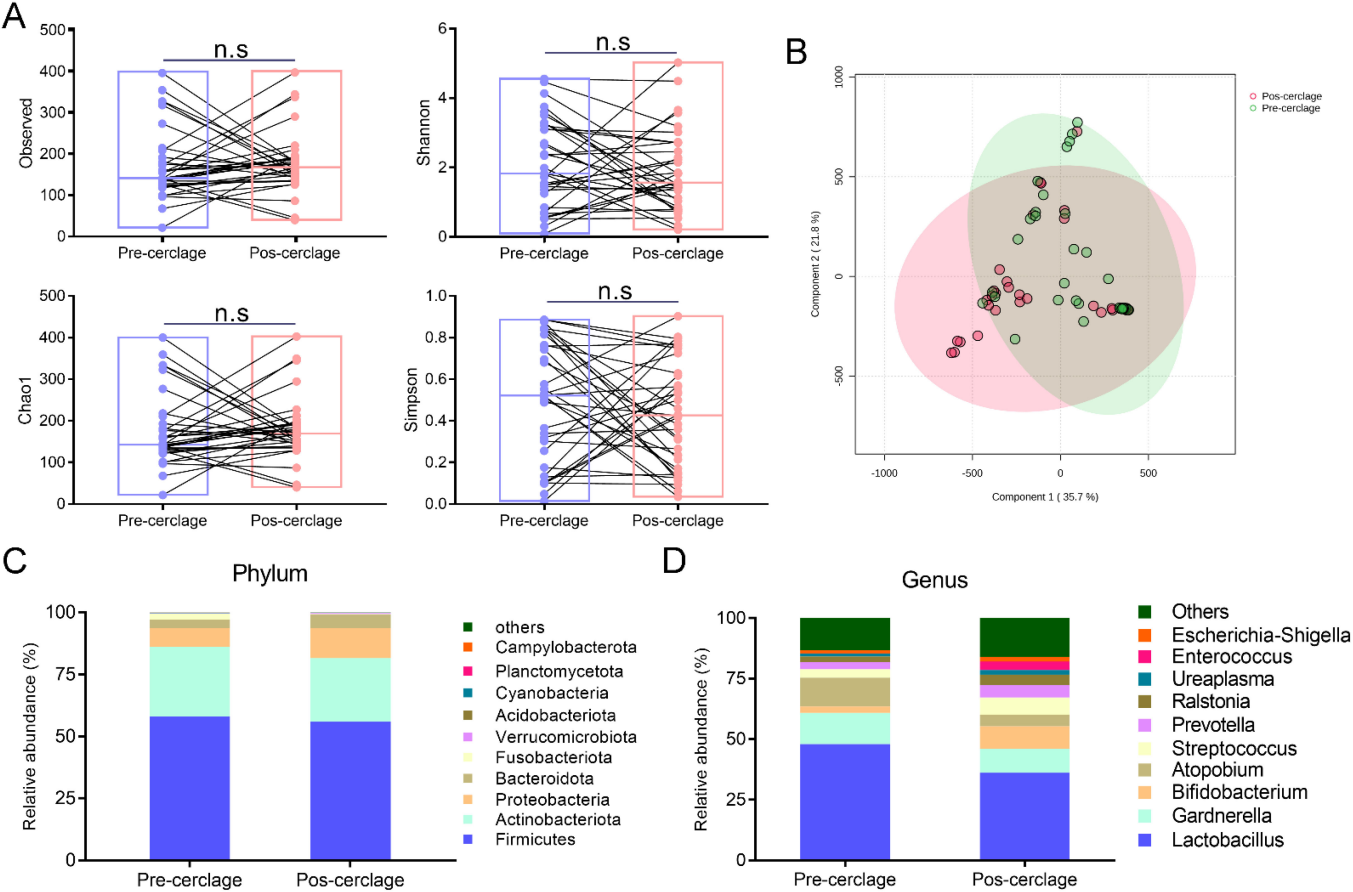
Effect of cervical cerclage on the community structure and diversity of vaginal microbiota. **(A)** α-diversity. **(B)** Principal component analysis (PCA). **(C)** The percentage of community abundance at the phylum level (top 10). **(D)** The percentage of community abundance at the genus level (Top 10). ns, not significant.

Vaginal microbial community perturbation by cervical cerclage was measured at different levels. To explore the influence of cervical cerclage on the diversity and composition of the vaginal microbiota during pregnancy, cervicovaginal lavage samples were collected from 35 participants before and after cervical cerclage, and their bacterial diversity was measured by 16S rRNA sequencing. As shown in **Figure 3A**, there was no significant difference in Observed (172.17 ± 84.25 vs. 177.43 ± 72.87), Shannon (2.09 ± 1.27 vs. 1.87 ± 1.16), Chao1 (0.49 ± 0.29 vs. 0.43 ± 0.26), and Simpson (174.52 ± 85.19 vs. 180.02 ± 74.14) indexes of the vaginal microbiota between pregnancy before and after cervical cerclage (*p* > 0.05). In addition, β-diversity was applied to observe the alteration in the vaginal microbiota between pregnancy before and after cervical cerclage. As shown in **Figure 3B**, the first and second principal components accounted for 35.7% and 21.8% of the total variables in the PCA plot score. Apparently, the composition of the vaginal microbiota was moved towards the positive direction of the first and second principal components, suggesting that cervical cerclage shifted the vaginal microbiota composition of pregnancy to a certain extent.

In detail, Firmicutes, Actinobacteriota, Proteobacteria, Bacteroidota, Fusobacteriota, Verrucomicrobiota, Acidobacteriota, Cyanobacteria, Planctomycetota, and Campylobacterota were the dominant vaginal microbiota during pregnancy before and after cervical cerclage at the phylum level (**Figure 3C**). At the genus level, *Lactobacillus*, *Gardberella*, *Bifidobacterium*, *Atopobium*, *Streptococcus*, *Prevotella*, *Ralstonia*, *Ureaplasma*, *Enterococcus*, and *Escherichia*-*Shigella* were the dominant vaginal microbiota during pregnancy before and after cervical cerclage (**Figure 3D**).

### 3.3 Specific bacteria enriched in the vagina during pregnancy after cervical cerclage

**Figure 4:**
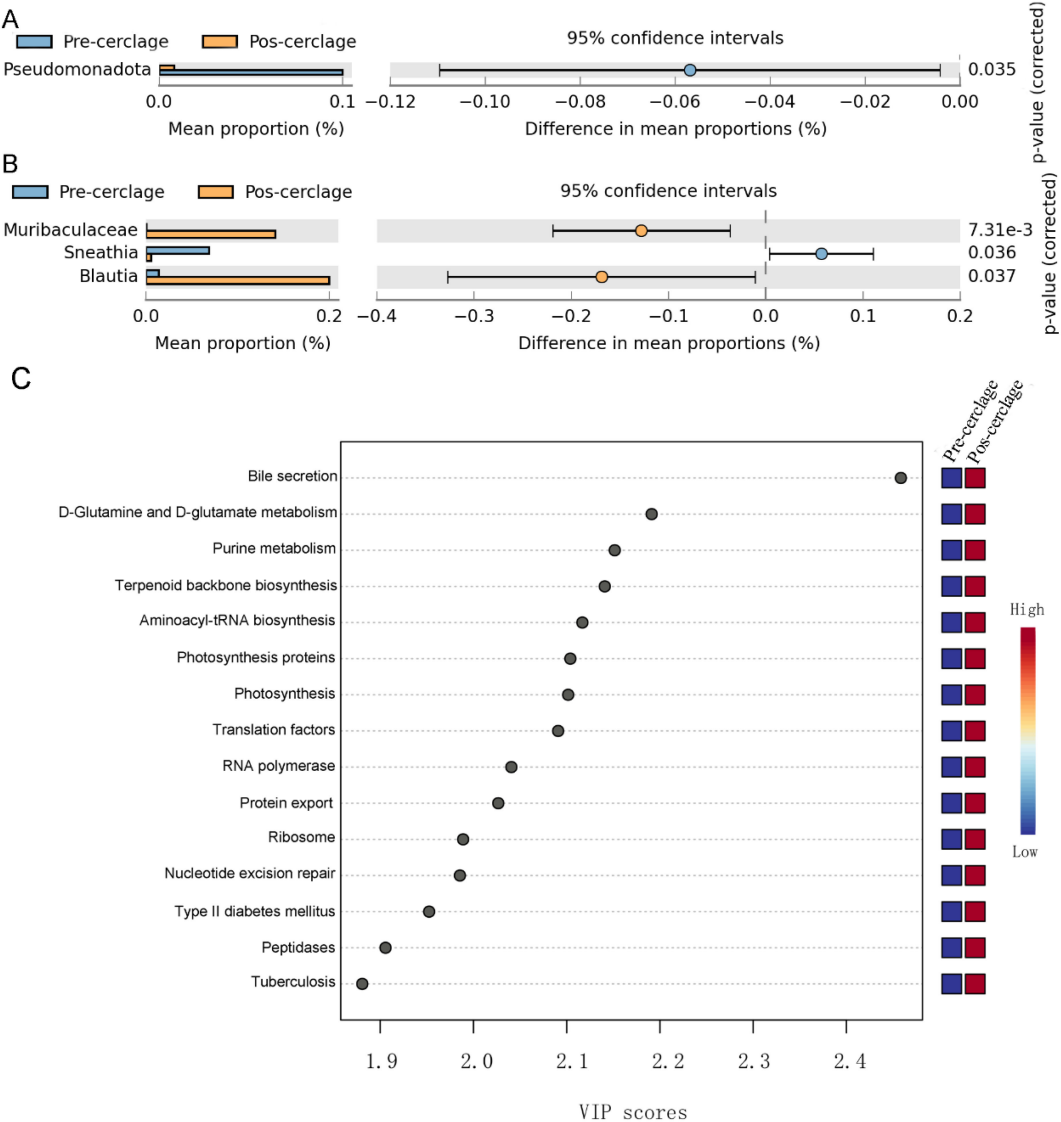
Effect of cervical cerclage on representative vaginal bacteria and their functions. **(A)** The present vaginal bacteria at the phylum level. **(B)** The present vaginal bacteria at the genus level. **(C)** The vaginal microbiota functional changes were predicted by PICRUSt2.

The bacteria with representative differences during pregnancy before and after cervical cerclage were determined by extended error bar analysis. At the phylum level, the relative abundance of Pseudomonadota was significantly reduced during pregnancy after cervical cerclage (**Figure 4A**). At the genus level, the relative abundance of *Muribaculaceae* and *Blautia* was significantly increased during pregnancy after cervical cerclage, but the relative abundance of *Sneathia* was significantly reduced (**Figure 4B**). The influence of cervical cerclage on the functionality of the vaginal microbiota was further explored (**Figure 4C**). Compared with the pre-cerclage group, the vaginal microbiota in the post-cerclage group was more closely associated with bile secretion, D-glutamine and D-glutamate metabolism, purine metabolism, terpenoid backbone biosynthesis, aminoacyl-tRNA biosynthesis, photosynthesis proteins, photosynthesis, translation factors, RNA polymerase, protein export, ribosome, nucleotide excision repair, type II diabetes mellitus, peptidases, and tuberculosis.

### 3.4 Effects of cervical cerclage on the metabolomics of vaginal microbes of patients

**Figure 5:**
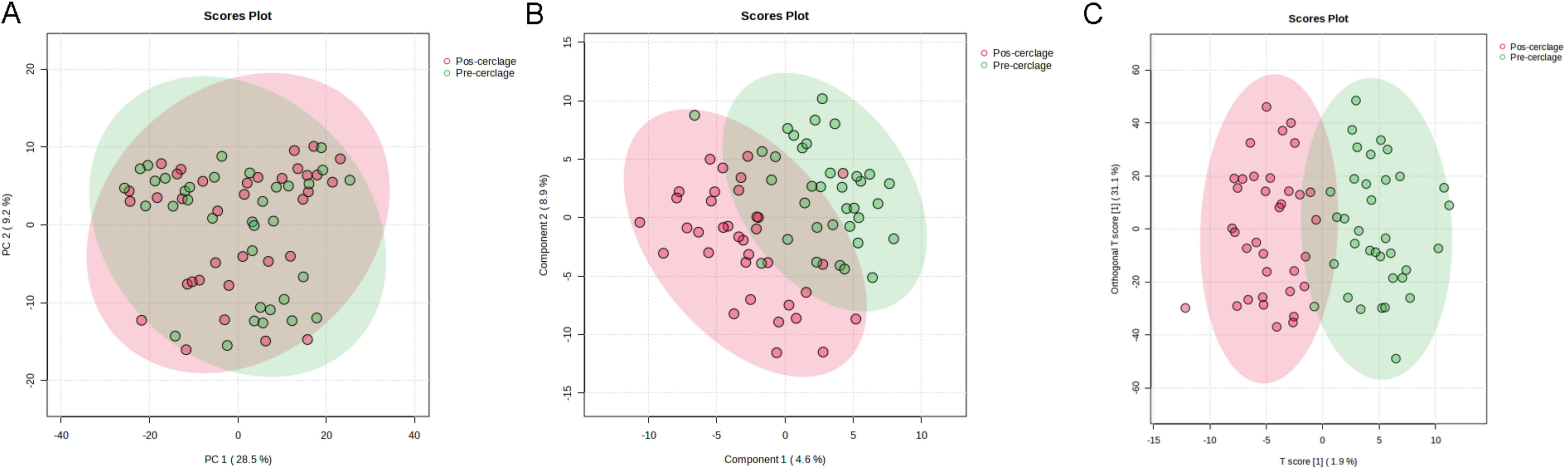
Effect of cervical cerclage on vaginal metabolites. **(A)** PCA; **(B)** PLS-DA; **(C)** OPLS-DA.

Metabolites in cervicovaginal lavage samples from pregnancy before and after cervical cerclage were further analyzed using untargeted metabolomics based on UPLC-Q-TOF/MS to reveal the therapeutic effects of cervical cerclage on pregnancy. As shown in **Figure 5A**, the results of the PCA plot score showed similar structures of vaginal metabolites between pregnancy before and after cervical cerclage. However, the results of PLS-DA and OPLS-DA showed that the sample from pregnancy after cervical cerclage was mainly distributed in the positive direction of the first principal components, indicating that cervical cerclage effectively altered the composition of vaginal metabolomics in pregnancy (**Figures 5B, C**).

### 3.5 Differential vaginal metabolites in pregnancy with cervical cerclage

**Figure 6:**
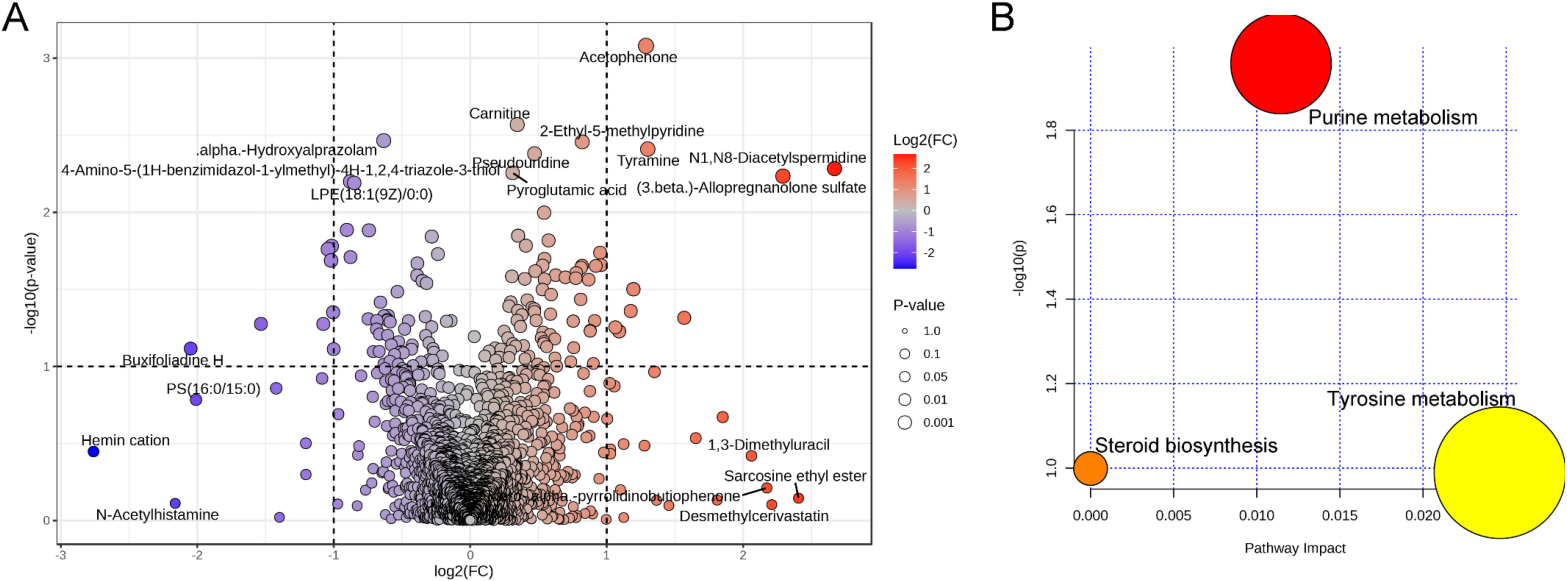
Effect of cervical cerclage on representative vaginal metabolites. **(A)** Volcanic composition of vaginal metabolites. **(B)** Analysis of the significant differential metabolites that were involved in the KEGG signaling pathway.

Overall, only a few alterations in vaginal metabolites during pregnancy after cervical cerclage were identified (**Figure 6A**). Compared with the pre-cerclage group, seven metabolites [namely, alpha-hydroxyalprazolam, 4-amino-5-(1H-benzimidazol-1-ylmethyl)-4H-1,2,4-triazole-3-thiol, LPE(18:1(9Z)/0:0), buxifoliadine H, PS(16:0/15:0), hemin cation, and *N*-acetylhistamine] were significantly downregulated in the post-cerclage group, and 12 metabolites [namely, acetophenone, carnitine, 2-ethyl-5-methylpyridine, pseudouridine, tyramine, N1,N8-diacetylspermidine, pyroglutamic acid, (3β)-allopregnanolone sulfate, 1,3-dimethyluracil, sarcosine ethyl ester, alpha-pyrrolidinobutiophenone, and desmethyl cerivastatin] were significantly upregulated. Finally, the results of differential material enrichment analysis showed that purine metabolism, steroid biosynthesis, and tyrosine metabolism were significantly altered in pregnancy after cervical cerclage (**Figure 6B**).

## 4 Discussion

Preterm delivery is defined as giving birth to an infant earlier than the 37th week of gestation, which is the most important cause of newborn illness and death. Cervical cerclage is an established intervention that is widely used in clinical settings, in order to prevent premature birth due to cervical insufficiency. Despite the success of cervical cerclage in decreasing the risk of preterm birth in singleton pregnancies, there were clear differences in the therapeutic efficacy of cervical cerclage among different individuals (Chen et al., 2024). In addition, the vaginal microbiota has received widespread attention worldwide and plays an essential role in the occurrence of preterm delivery (Glover et al., 2019). Therefore, we speculated that the therapeutic efficacy of cervical cerclage may be associated with the composition of the vaginal microbiota and its metabolites. In the present study, 16S rRNA gene sequencing, untargeted metabolome, and multiple statistical analyses were used to detect and screen the key vaginal microbiota and differential vaginal metabolites during pregnancy after cervical cerclage.

Although the study of the vaginal microbiota is relatively small compared with that of the gut microbiota, the vaginal microbiota accounts for approximately 9% of the total human microbiota, which plays an essential role in improving the vagina’s health. The vaginal microbiota is a dynamic ecosystem composed of various microorganisms with different quantities and ratios, which maintains the integrity of the vaginal barrier and prevents the growth of harmful bacteria. A previous study showed that abnormalities in the vaginal microbiota have been associated with the occurrence of preterm birth. The α-diversity of the vaginal microbiota in healthy women was obviously lower than that in women with vaginal diseases, which is different from the gut microbiota. In addition, the α-diversity of the vaginal microbiota is also frequently used to establish the prediction model of gestational age at delivery. In this study, the α-diversity (namely, Observed, Shannon, Chao1, and Simpson indexes) of the vaginal microbiota was not significantly altered during pregnancy after cervical cerclage, indicating that the therapeutic efficacy of cervical cerclage is not associated with the alteration in α-diversity of the vaginal microbiota.

The Bacteroidetes phylum (includes Gram-negative bacteria) and the Firmicutes phylum (includes Gram-positive and low G + C-type bacteria) are widely distributed in the vagina. In the intestine, studies have shown that the proportion of Firmicutes and Bacteroidetes was closely related to the occurrence of hyperglycemia and hyperlipidemia. In this study, we found that the reduction of the vaginal Firmicutes phylum and the increase of the vaginal Bacteroidetes phylum during pregnancy after cervical cerclage may be beneficial for reducing the risk of hyperglycemia and hyperlipidemia during pregnancy. Acidimicrobiales is a group of planktonic Actinobacteriota that is often overlooked in the vagina and gut. The association between the alteration in Acidimicrobiales abundance and the host’s health was only determined indirectly using correlation analysis. At the genus level, *Lactobacillus* and *Gardnerella* appear to be predominant in the vaginal microbiota. *Lactobacillus* is regarded as a probiotic that is beneficial for the host’s health when given in adequate amounts. *Lactobacillus* processes a series of physiological effects, such as suppressing oxidative stress and inflammatory responses (Qin et al., 2024). *Lactobacillus* also prevents the growth of harmful bacteria in the vagina by elevating the levels of short-chain fatty acids (Guo et al., 2023a). The lower abundance of *Lactobacillus* in the vagina has been confirmed to be closely associated with the high risk of preterm birth (Jothi et al., 2024). In the present study, we also found that the relative abundance of *Lactobacillus* is positively related to gestational age at delivery. However, *Gardnerella* is widely distributed in women of childbearing age, and the high proportion of *Gardnerella* causes a series of diseases, which is extensively used to establish the bacterial vaginitis model (Jothi et al., 2024). Meanwhile, *Gardnerella* can produce sialic acidase and proline aminopeptidase that destroy the protective factors in the vagina and facilitate the adhesion of anaerobic bacteria on the vaginal mucosa surface. *Gardnerella* can promote the growth and reproduction of pathogenic bacteria by producing massive amounts of amines, which further elevates the risk of preterm birth (Gao et al., 2021). In addition, *Bifidobacterium* belongs to the phylum Actinobacteria, which is one of the most extensively studied and utilized probiotic bacteria. Oral administration of *Bifidobacterium* suppressed the growth of harmful bacteria, oxidative stress, and inflammatory responses. A previous study also found that *Bifidobacterium* intervention prevents the development of inflammatory responses by regulating the gut microbiota (Guo et al., 2023b). In this study, the relative abundance of *Lactobacillus* was downregulated during pregnancy after cervical cerclage, but the relative abundance of *Gardnerella* and *Bifidobacterium* was upregulated, possibly due to cervical cerclage that reduced the oxygen content in the vagina, which is beneficial for promoting the growth of anaerobic bacteria (*Gardnerella* and *Bifidobacterium*). The rapid reproduction of anaerobic bacteria further suppressed the growth of facultative anaerobic bacteria by striving for nutrient substance and ecological niche.

Pseudomonadota are extensively distributed in the human oral cavity, stomach, gut, skin, and vagina. Pseudomonadota includes some of the most prevailing bacterial groups and a series of opportunistic pathogens that is difficult to inactivate with existing antibacterial drugs (Leão et al., 2023). A previous report suggested that a high abundance of Pseudomonadota induces the occurrence of inflammatory responses, which promotes the occurrence of some diseases (Zhang et al., 2020). In this study, the relative abundance of Pseudomonadota in the post-cerclage group was higher than that in the pre-cerclage group. In addition, *Muribaculaceae* have attracted a great deal of attention worldwide because they play the most important role in improving the host’s health (Guo et al., 2025). *Muribaculaceae* have the ability to metabolize polysaccharides into short-chain fatty acids that provide energy for the growth of epithelial cells, which is beneficial for maintaining the integrity of the barrier (Qiu et al., 2024). A previous study found that the relative abundance of *Muribaculaceae* in patients was higher than that in healthy people (Efremova et al., 2024). *Blautia* (belonging to the Lachnospiraceae family) is widely prevalent in the feces, intestines, and vagina of mammals, and their abundance has been influenced by a series of factors, especially diet and age. *Blautia* intervention effectively inhibited the production of pro-inflammatory cytokines and oxidative stress by regulating TLR4/NF-κB and Nrf2/HO-1 pathways, respectively (Mao et al., 2024). However, *Sneathia* act as an opportunistic pathogen that is commonly found in the reproductive tract and have been associated with bacterial vaginosis. The high abundance of *Sneathia* may increase the occurrence of preterm birth by promoting the release of pro-inflammatory cytokines and destroying the vaginal barrier (Panzer et al., 2025). A previous study showed that the abundance of *Sneathia* is positively associated with the occurrence of preterm birth (Talukdar et al., 2024). In this study, we found that cervical cerclage effectively increased the relative abundance of vaginal *Muribaculaceae* and *Blautia*, as well as reduced the relative abundance of vaginal *Sneathia* during pregnancy, suggesting that cervical cerclage suppressed the growth of harmful bacteria and inflammatory responses by regulating the vaginal microbiota composition. Therefore, we speculate that the inhibition of *Sneathia* growth may be beneficial for reducing the risk of preterm birth.

As is well known, the alteration of gut microbiota composition directly affects gut metabolites. Therefore, we speculated that the vaginal microbiota is the most important factor that causes changes in vaginal metabolites. Compared with the pre-cerclage group, the concentration of alpha-hydroxyalprazolam, LPE (18:1(9Z)/0:0), PS(16:0/15:0), and *N*-acetylhistamine was significantly decreased in the post-cerclage group. Alpha-hydroxyalprazolam is a metabolite of both alprazolam and adinazolam, which have anxiolytic, anticonvulsant, sedative, and antidepressant properties (Abarca and Gerona, 2023). LPE serves as the most important component of the cell membrane, which is easily derived from phosphatidylethanolamine by deacylation by phospholipase A2 (Dai et al., 2024). The increases in LPE concentration may be associated with cell injury. PS (16:0/15:0) is a phosphatidylserine, and its concentrations directly reflect the degree of vaginal contamination. *N*-acetylhistamine is an intermediate in histidine metabolism that is strongly related to the formation of blood stasis syndrome (Yinxing et al., 2023). However, carnitine consists of the amino acids lysine and methionine that promote fatty acid transformation into mitochondria and accelerate the accumulation of ATP by the β-oxidation of fatty acid, which is beneficial for reducing the risk of preterm birth (Manuck et al., 2021). Pseudouridine, also known as “the fifth nucleotide,” is estimated to comprise ∼5% of the total of all cellular RNA nucleotides, which promote the production of reticulocyte counts and hematocrits (Cerneckis et al., 2022). Pyroglutamic acid (5-oxoproline) is a cyclized derivative of L-glutamic acid, and it is reported that its concentration was significantly reduced in alcohol-induced liver injury mice (Huo et al., 2020). Higher amino acid metabolite levels representing increased vaginal amino acid utilization are closely associated with vaginosis-related bacteria, which may use vaginal amino acids as carbon and nitrogen sources to support their growth, which may elevate the risk of preterm birth (Zhang et al., 2022). Therefore, the high level of amino acid metabolites or the low level of amino acid in the vagina may act as a marker of preterm birth. Allopregnanolone sulfate serves as one of the steroid hormones, and its contents in the vagina are strongly associated with spontaneous preterm birth (Syan et al., 2017). In this study, cervical cerclage effectively elevated the concentration of vaginal carnitine, pseudouridine, and allopregnanolone in pregnancy. In addition, we found that the purine metabolism was significantly upregulated during pregnancy after cervical cerclage. A previous study suggested that the downregulation of purine metabolism induced the occurrence of inflammatory responses, accelerating inflammation-triggered cervical softening (Mager et al., 2020). Therefore, upregulation of purine metabolism is beneficial in reducing the risk of preterm birth by suppressing the inflammatory responses and maintaining the integrity of the intestinal barrier.

## 5 Conclusion

In summary, the clinical characteristics and α-diversity of the vaginal microbiota were not significantly changed during pregnancy after cervical cerclage. However, the vaginal microbiota composition and its metabolites were obviously altered during pregnancy after cervical cerclage, which may be involved in purine metabolism and amino acid metabolism. These results have prominent implications for ensuring cervical cerclage supplementation during pregnancy and preventing adverse outcomes. Although our findings offer preliminary insights into specific topics, the conclusions warrant cautious interpretation owing to the limited sample size.

## Data Availability

The datasets presented in this study can be found in online repositories. The names of the repository/repositories and accession number(s) can be found below: https://www.ncbi.nlm.nih.gov/, PRJNA1122359.

## References

[B1] Abarca R. Gerona R. (2023). Development and validation of an LC-MS/MS assay for the quantitative analysis of alprazolam, α-hydroxyalprazolam and hydrocodone in dried blood spots. J. Chromatogr. B 1220, 123639. doi: 10.1016/j.jchromb.2023.123639 36906954

[B2] Beernink R. H. J. Schuitemaker J. H. N. Zwertbroek E. F. Scherjon S. A. Cremers T. I. (2023). Early pregnancy biomarker discovery study for spontaneous preterm birth. Placenta 139, 112–119. doi: 10.1016/j.placenta.2023.06.011 37356366

[B3] Cerneckis J. Cui Q. He C.. (2022). Decoding pseudouridine: an emerging target for therapeutic development. Trends Pharmacol. Sci. 43, 522–535. doi: 10.1016/j.tips.2022.03.008 35461717

[B4] Chen C. Guo S. Fan C. Guo F. (2024). Nomogram-based risk assessment for emergency cervical cerclage failure in patients with cervical insufficiency. Heliyon 10, e32923. doi: 10.1016/j.heliyon.2024.e32923 39027507 PMC11255580

[B5] Chen R. Peng C. Wang Z. Xiao Y. Tang S. (2024). Effects of vaginal microbiota on human papillomavirus infection and its related diseases. Microbial Pathogenesis 193, 106761. doi: 10.1016/j.micpath.2024.106761 38925345

[B6] Dai M. Shi Q. Xiang X. Zhao X. Zeng Z. Jin S.. (2024). Metabolomics study of lipid lowering effect and lysophospholipids regulation by Alismatis rhizoma and processed forms in hyperlipidemia mice. Chin. J. Analytical Chem. 52, 100431. doi: 10.1016/j.cjac.2024.100431

[B7] Efremova I. Maslennikov R. Medvedev O. Kudryavtseva A. Avdeeva A. Krasnov G.. (2024). Gut microbiota and biomarkers of intestinal barrier damage in cirrhosis. Microorganisms 12, 463. doi: 10.3390/microorganisms12030463 38543514 PMC10972037

[B8] Elkhouli M. Raghuram K. Elhanafy T. Asztalos E. Banihani R. Shah P.S.. (2024). Association of low hemoglobin at birth and neurodevelopmental outcomes in preterm neonates ≤28 weeks’ gestation: a retrospective cohort study. J. Perinatology 44, 880–885. doi: 10.1038/s41372-024-01946-y 38553601

[B9] Fettweis J. M. Serrano M. G. Brooks J. P. Edwards D. J. Girerd P. H. Parikh H. I.. (2019). The vaginal microbiome and preterm birth. Nat. Med. 25, 1012–1021. doi: 10.1038/s41591-019-0450-2 31142849 PMC6750801

[B10] Gao Y. Shang Q. Wei J. Chen T. (2021). The correlation between vaginal microecological dysbiosis-related diseases and preterm birth: A review. Med. Microecology 8, 100043. doi: 10.1016/j.medmic.2021.100043

[B11] Glover A. V. Chi L. Lu K. Manuck T. (2019). 523: Distinct cervicovaginal space microbiota are associated with SPTB in women undergoing cervical cerclage. Am. J. Obstetrics Gynecology 220, S350–S351. doi: 10.1016/j.ajog.2018.11.545

[B12] Guo W. Cui S. Tang X. Yan Y. Xiong F. Zhang Q.. (2023b). Intestinal microbiomics and hepatic metabolomics insights into the potential mechanisms of probiotic *Bifidobacterium pseudolongum* CCFM1253 preventing acute liver injury in mice. J. Sci. Food Agric. 103, 5958–5969. doi: 10.1002/jsfa.v103.12 37099000

[B13] Guo W. Cui S. Tang X. Zhang Q. Zhao J. Mao B.. (2023a). Intestinal microbiomics and metabolomics insights into the hepatoprotective effects of *Lactobacillus paracasei* CCFM1222 against the acute liver injury in mice. Probiotics Antimicrobial Proteins 15, 1063–1077. doi: 10.1007/s12602-022-09986-6 36056292

[B14] Guo W. Liu W. Liang P. Ni L. Lv X. Fan J.. (2025). High molecular weight polysaccharides from Ganoderma lucidum attenuates inflammatory responses, gut microbiota, and liver metabolomic in lipopolysaccharide-induced liver injury mice. Int. J. Biol. Macromolecules 287, 138400. doi: 10.1016/j.ijbiomac.2024.138400 39657883

[B15] Hong X. Surkan P. J. Zhang B. Keiser A. Ji Y. Ji H.. (2021). Genome-wide association study identifies a novel maternal gene × stress interaction associated with spontaneous preterm birth. Pediatr. Res. 89, 1549–1556. doi: 10.1038/s41390-020-1093-1 32726798 PMC8400921

[B16] Huo T. G. Fang Y. Zhang Y. H. Feng C. Jiang H. (2020). Liver metabonomics study on the protective effect of glycyrrhetinic acid against realgar-induced liver injury. Chin. J. Natural Medicines 18, 138–147. doi: 10.1016/S1875-5364(20)30014-5 32172949

[B17] Jothi R. Kamaladevi A. Muthuramalingam P. Malligarjunan N. Pandian S. K. Gowrishankar. S. (2024). Untargeted metabolomics uncovers prime pathways linked to antibacterial action of citral against bacterial vaginosis-causing Gardnerella vaginalis: An *in vitro* and *in vivo* study. Heliyon 10, e27983. doi: 10.1016/j.heliyon.2024.e27983 38545203 PMC10966606

[B18] Leão I. de Carvalho T. B. Henriques V. Ferreira C. Sampaio-Maia B. Manaia C. M. (2023) Pseudomonadota in the oral cavity: a glimpse into the environment-human nexus. Appl. Microbiol. Biotechnol. 107, 517–534. doi: 10.1007/s00253-022-12333-y 36567346 PMC9842593

[B19] Li L. Huang X. Yan J. Zhang J. Yang D. Pan M. (2023). Predictive value of serum inflammatory markers for histological chorioamnionitis among women with preterm premature rupture of membranes after undergoing cervical cerclage. Clinics 78, 100292. doi: 10.1016/j.clinsp.2023.100292 37879248 PMC10618699

[B20] Liao J. Shenhav L. Urban J. A. Serrano M. Zhu B. Buck G. A.. (2023). Microdiversity of the vaginal microbiome is associated with preterm birth. Nat. Commun. 14, 4997. doi: 10.1038/s41467-023-40719-7 37591872 PMC10435516

[B21] Mager L. F. Burkhard R. Pett N. Cooke N. C. A. Brown K. Ramay H.. (2020). Microbiome-derived inosine modulates response to checkpoint inhibitor immunotherapy. Sci. (New York N.Y.) 369, 1481–1489. doi: 10.1126/science.abc3421 32792462

[B22] Manuck T. A. Lai Y. Ru H. Rager J. E. Fry R. C. Lu K.. (2021). Metabolites from midtrimester plasma of pregnant patients at high risk for preterm birth. Am. J. Obstetrics Gynecology MFM 3, 100393. doi: 10.1016/j.ajogmf.2021.100393 PMC8555706 33991707

[B23] Mao B. Guo W. Cui S. Zhang Q. Zhao J. Tang X.. (2024). *Blautia producta* displays potential probiotic properties against dextran sulfate sodium-induced colitis in mice. Food Sci. Hum. Wellness 13, 709–720. doi: 10.26599/FSHW.2022.9250060

[B24] Ohuma E. O. Moller A. B. Bradley E. Chakwera S. Hussain-Alkhateeb L. Lewin A.. (2023). National, regional, and global estimates of preterm birth in 2020, with trends from 2010: A systematic analysis. Lancet 402, 1261–1271. doi: 10.1016/S0140-6736(23)00878-4 37805217

[B25] Panzer J. J. McGinnis N. Blom J. Winters A. D. Sobel J. D. Theis K. R.. (2025). Draft genomes of three Sneathia vaginalis isolates from a patient with bacterial vaginosis. Microbiol. Resource Announcements 14, e0094124. doi: 10.1128/mra.00941-24 PMC11895484 39936913

[B26] Peelen M. J. C. S. Luef B. M. Lamont R. F. de Milliano I. Jensen J. S. Limpens J.. (2019). The influence of the vaginal microbiota on preterm birth: A systematic review and recommendations for a minimum dataset for future research. Placenta 79, 30–39. doi: 10.1016/j.placenta.2019.03.011 31047708

[B27] Qin J. Ma Y. Wang C. Li H. Zhou Z. Zhang Y.. (2024). Effects of carnosine combined with Lactobacillus on the antioxidant capacity of liver and kidney in normal or stressed mice. Food Bioscience 59, 103904. doi: 10.1016/j.fbio.2024.103904

[B28] Qiu L. Yan C. Yang Y. Liu K. Yin Y. Zhang Y.. (2024). Morin alleviates DSS-induced ulcerative colitis in mice via inhibition of inflammation and modulation of intestinal microbiota. Int. Immunopharmacol. 140, 112846. doi: 10.1016/j.intimp.2024.112846 39121607

[B29] Schuster H. J. Bos A. M. Himschoot L. Savelkoul P. H. M. Painter R. C. van Houdt R.. (2024). Vaginal microbiota and spontaneous preterm birth in pregnant women at high risk of recurrence. Heliyon 10, e30685. doi: 10.1016/j.heliyon.2024.e30685 38803950 PMC11128838

[B30] Seyama R. Makino S. Takeda J. Takeda S. Itakura A. (2022). The retrospective study for effectiveness of cervical cerclage in preventing recurrent preterm birth. Taiwanese J. Obstetrics Gynecology 61, 63–69. doi: 10.1016/j.tjog.2021.11.012 35181048

[B31] Syan S. K. Minuzzi L. Costescu D. Coote M. Hall G. B. C. Frey B. N.. (2017). Influence of endogenous estradiol, progesterone, allopregnanolone, and dehydroepiandrosterone sulfate on brain resting state functional connectivity across the menstrual cycle. Fertility Sterility 107, 1246–1255.e4. doi: 10.1016/j.fertnstert.2017.03.021 28476183

[B32] Talukdar D. Sarkar M. Ahrodia T. Bhatnagar S. Mukherjee S. Das B.. (2024). Previse preterm birth in early pregnancy through vaginal microbiome signatures using metagenomics and dipstick assays. iScience 27, 111238. doi: 10.1016/j.isci.2024.111238 39569373 PMC11576381

[B33] Wang L. Di J. Wang Q. Zhang H. Zhao W. Shi X.. (2024). Heat exposure induced risks of preterm birth mediated by maternal hypertension. Nat. Med. 30, 1974–1981. doi: 10.1038/s41591-024-03002-w 38750350

[B34] Wei J. Zhang L. Xu H. Lu Q. (2024). Preterm birth, a consequence of immune deviation mediated hyperinflammation. Heliyon 10, e28483. doi: 10.1016/j.heliyon.2024.e28483 38689990 PMC11059518

[B35] Yinxing L. Zijun C. Yiqin W. Xihua C. Jie L. Lingli C.. (2023). Mechanisms of Yangxin Tongmai Formula for blood stasis syndrome in coronary heart disease rats based on untargeted plasma metabolomics and intestinal flora 16S rRNA sequencing. Digital Chin. Med. 6, 198–209. doi: 10.1016/j.dcmed.2023.07.009

[B36] Zhang Q. Chen R. Li M. Zeng Z. Zhang L. Liao Q.-p.. (2022). The interplay between microbiota, metabolites, immunity during BV. Med. Microecology 11, 100049. doi: 10.1016/j.medmic.2021.100049

[B37] Zhang T. Wang Y. Yan W. Tao Y. Jia J. Cai W.. (2020). Microbial alteration of small bowel stoma effluents and colonic feces in infants with short bowel syndrome. J. Pediatr. Surg. 55, 1366–1372. doi: 10.1016/j.jpedsurg.2019.08.004 31493882

